# A Technology-Mediated Behavioral Weight Gain Prevention Intervention for College Students: Controlled, Quasi-Experimental Study

**DOI:** 10.2196/jmir.5474

**Published:** 2016-06-13

**Authors:** Delia Smith West, Courtney M Monroe, Gabrielle Turner-McGrievy, Beth Sundstrom, Chelsea Larsen, Karen Magradey, Sara Wilcox, Heather M Brandt

**Affiliations:** ^1^ Arnold School of Public Health University of South Carolina Columbia, SC United States; ^2^ Department of Communication College of Charleston Charleston, SC United States

**Keywords:** weight gain prevention, college students, social media, wearables, behavior change

## Abstract

**Background:**

Both men and women are vulnerable to weight gain during the college years, and this phenomenon is linked to an increased risk of several chronic diseases and mortality. Technology represents an attractive medium for the delivery of weight control interventions focused on college students, given its reach and appeal among this population. However, few technology-mediated weight gain prevention interventions have been evaluated for college students.

**Objective:**

This study examined a new technology-based, social media-facilitated weight gain prevention intervention for college students.

**Methods:**

Undergraduates (*n* =58) in two sections of a public university course were allocated to either a behavioral weight gain prevention intervention (Healthy Weight, HW; N=29) or a human papillomavirus (HPV) vaccination awareness intervention (control; N=29). All students were enrolled, regardless of initial body weight or expressed interest in weight management. The interventions delivered 8 lessons via electronic newsletters and Facebook postings over 9 weeks, which were designed to foster social support and introduce relevant educational content. The HW intervention targeted behavioral strategies to prevent weight gain and provided participants with a Wi-Fi-enabled scale and an electronic physical activity tracker to facilitate weight regulation. A repeated-measures analysis of variance was conducted to examine within- and between-group differences in measures of self-reported weight control practices and objectively measured weight. Use of each intervention medium and device was objectively tracked, and intervention satisfaction measures were obtained.

**Results:**

Students remained weight stable (HW: −0.48+1.9 kg; control: −0.45+1.4 kg), with no significant difference between groups over 9 weeks (*P* =.94). However, HW students reported a significantly greater increase in the number of appropriate weight control strategies than did controls (2.1+4.5 vs −1.1+3.4, respectively; *P* =.003) and there was no increase in inappropriate weight control behaviors (*P* =.11). More than 90% of students in the HW arm opened the electronic newsletters each week, and the average number of Facebook interactions (comments and likes) per student each week was 3.3+1.4. Each self-monitoring device was initialized by 90% of HW students. On average, they used their physical activity tracker for 23.7+15.2 days and their Wi-Fi scale for 14.1+13.1 days over the 9 weeks. HW students rated the intervention favorably.

**Conclusions:**

The short-term effect of this technology-based weight gain prevention intervention for college students is promising and merits evaluation over a longer duration to determine whether engagement and behavioral improvements positively affect weight outcomes and can be maintained.

## Introduction

Young adulthood represents a period in which weight gain and the onset of obesity are common [1,2], and these patterns are associated with an increased risk of chronic diseases and mortality [3-5]. The phenomenon of weight gain among college freshmen in particular is well known, with one recent meta-analysis reporting increases in weight ranging from 0.73 to 3.99 kg and an average weight gain of 1.74 kg among studies which objectively measured students’ weight [6]. Furthermore, weight gain continues throughout the full college period, and both men and women are vulnerable to this pattern [7-10]. Of note, an estimated one-third of college students are overweight or obese [11]. These facts are especially concerning given that college represents a critical transition when young adults begin to make independent decisions about a range of personal choices, including lifestyle behaviors [1]. The physical activity and dietary patterns they adopt—the two key lifestyle factors influencing weight [12]—will likely track into adulthood [1]. It is evident that the college years present a prime target period in which effective weight management should be promoted in an effort to shape the future health and well-being of a large number of individuals.

Technology offers an attractive platform for behavioral weight control interventions targeting college students because it is both familiar and appealing to young adults. The vast majority of college students regularly use the Internet (99%) [13] and mobile phones (80%) [14] to access information and connect socially, particularly through social networking platforms. Most college students (80%) use online social networks [13]; Facebook is the most widely used social networking platform among individuals aged between 18 and 29 years [15]. Furthermore, advanced technologies, such as “wearables” (eg, electronic physical activity trackers) and electronically-enabled health monitoring devices (eg, Wi-Fi body weight scales), are increasingly available and hold promise for managing weight [16-18]. These technologies provide opportunities for both collecting objective data and delivering intervention components in real-time, bringing interventions to the setting in which behaviors occur.

Interventions to promote healthy diet [19-23], physical activity [24-28], and weight control [29-33] among college students have shown potential. However, only a small number of studies have evaluated the effectiveness of technology-based interventions designed specifically for weight gain prevention among college students [34-36], and the findings from these studies have been mixed. There is a clear need for the continued development and evaluation of technology-based weight gain prevention interventions targeting college students. Indeed, in a recent review of the limited body of research on weight gain prevention interventions in college students, Laska et al [37] called for the further design and evaluation of interventions that capitalize on the crucial influences of technology and social networks for this population. Importantly, no healthy weight interventions targeting college students have sought to harness the joint capabilities of advanced technological monitoring devices and online social networks. The purpose of this study was to examine a novel, technology-mediated weight gain prevention intervention for college students.

## Methods

### Study Design and Procedures

This controlled, quasi-experimental study recruited undergraduates enrolled in two sections of an advanced health communication class at a public university in the Southeastern United States. Baseline questionnaires were administered via a secure website (Qualtrics, Provo, Utah), and anthropometric data were collected in-person by the study staff. Both classes received an 8-session, technology-mediated health promotion intervention that provided health education and facilitated social support, delivered over 9 weeks. Classes were randomly allocated by a coin toss to either (1) a behavioral weight gain prevention intervention (Healthy Weight, HW) or to (2) a human papillomavirus (HPV) vaccination awareness intervention (control). Each health promotion intervention served as a control for the other. This study design allowed for the simultaneous implementation and evaluation of interventions centered on two areas of health promotion that are particularly relevant to college students.

The HW intervention focused on weight gain patterns among college populations and long-term risk for obesity-related chronic diseases, as well as behavioral strategies to maintain weight and avoid obesity. Participants in the HW intervention were given a Wi-Fi-enabled scale (Aria; Fitbit Inc., Boston, MA) and an electronic physical activity tracker (Fitbit Zip; Fitbit Inc., Boston, MA) to facilitate weight regulation. Participants in both interventions were emailed weekly electronic newsletters targeting relevant content and enrolled in separate, private Facebook groups. These groups served as an additional channel for the delivery of intervention content, as well as promoted interaction between fellow group members, facilitated by study counselors. The control condition did not include any healthy weight-related behavior change elements. The interventions were matched for intervention duration and structure, as well as the number of newsletters and planned Facebook content postings. Newsletters were sent automatically via email using MailChimp (Rocket Science Group, LLC, Atlanta, GA), which allows prescheduling of distribution and also tracks who opens the newsletter link; both groups received newsletters on the same schedule. Posttreatment questionnaire data and body weight measurements were obtained 9 weeks after intervention initiation. Detailed descriptions of specific measures administered are given below. Primary outcomes for the HW intervention described here were change in body weight and in self-reported weight control behaviors. The Institutional Review Boards at both the College of Charleston and University of South Carolina approved the study.

### Participant Recruitment and Eligibility

Email invitations outlining the study and eligibility criteria were sent to students enrolled in the targeted courses. Each student could voluntarily elect to either participate in the study as a course assignment or engage in an alternative course activity. To be included, students were required to be registered for the course, have access to the Internet via a computer and/or mobile device, and be willing to use their existing email address and Facebook account, or establish a new email address and Facebook account, for the study. Students were also required to provide informed consent on a secure website.

### Healthy Weight Intervention

The HW intervention was based on the social cognitive theory (SCT) [38] and content was adapted from the Diabetes Prevention Program Lifestyle Intervention [39] and technology-mediated behavioral weight control programs [18,40,41]. The content was delivered by weekly electronic newsletters and Facebook postings coupled with technological tools that provided objective data on physical activity and body weight. Participants were encouraged to weigh themselves daily using the Wi-Fi scale provided and to track their weight over time using the affiliated website or mobile app. The rationale for daily weighing was provided and a self-regulation approach to promote weight maintenance and stability by initiating suitable weight management behaviors when body weight increased [18,42] was advocated. Daily self-weighing has been shown to facilitate weight loss and maintenance [43,44], with no evidence of harms associated with the practice [45,46]. Participants were given methods to identify their current weight status and information that directed those who might be overweight to engage in more focused weight management efforts, whereas those who were not overweight were instructed to focus their efforts on making their dietary intake and physical activity levels healthier while maintaining their current weight.

A key strategy for promoting healthy weight and fostering positive lifestyle habits was to emphasize increased physical activity. The electronic physical activity tracker provided personalized and real-time objective feedback on steps taken and miles walked, as well as cumulative personal reports on activity level, via the associated website and mobile app. Participants were given graded goals to increase their steps to at least 10,000/day [47] and suggestions were offered about how to use the feedback from the tracker to set proximal goals and stay motivated. Campus walking routes and university gym hours were provided in lesson materials to facilitate adoption of increased physical activity.

Healthy dietary intake patterns were addressed in the intervention, and behavioral strategies for improving diet quality were offered. Facebook posts and newsletters focused on building social support for selecting more fruits and vegetables, lower-fat dairy products, and lower-calorie and/or more nutrient-dense snack options, as well as validating a social norm around eating a healthier diet. However, no specific calorie goals or food patterns were prescribed.

Lesson content focused on themes relevant to college students engaged in healthy weight regulation (Table 1). Each lesson was tailored specifically for the campus, with examples of locations or events that promoted physical activity and tips on healthy food choices in the dining halls and local restaurants. In addition, study personnel posted at least five posts each week to the private Facebook message board. Posts were scheduled to allow automatized distribution using a social media management tool (Hootsuite Media Inc., Vancouver, BC, Canada). Study investigators moderated the Facebook page, providing answers to questions or stimulating interaction, as appropriate. The posts were designed to cultivate a social climate of support for behavior change and included questions designed to prompt discussion about behavior change efforts, encourage social support from peers for new health behaviors, model successful weight control behaviors, and elicit suggestions to overcome barriers encountered. Polls were used to provide social norming for healthy behaviors. These types of messages have been shown to stimulate discussion more effectively during social media–delivered weight loss interventions [40]. Intervention activities (reading the newsletters and interacting on Facebook) were intended to take approximately 30 min/week in total. The program spanned a 9-week period during the spring semester, inclusive of spring break.

**Table 1:** Lesson topics delivered via electronic newsletters and Facebook posts.

Session	Topics
1	Introduction to Healthy Weight intervention, setting up monitoring devices, and on-campus resources
2	Energy balance, self-monitoring, goal setting, and overcoming barriers
3	Nutrition and healthy dietary intake practices
4	Physical activity benefits and recommendations
5	Healthy eating and physical activity specific to the university setting
6	Sugar sweetened beverages, dining out, stress, and alcohol
7	Relapse prevention and social support
8	Overview of healthy weight control practices

### Control Intervention

The control group received a HPV vaccination awareness intervention with similar elements to those implemented in the HW intervention. In brief, the control intervention was intended to increase knowledge about the health consequences of HPV and the benefits of the vaccination series, which can prevent certain types of HPV. Over the 9-week period, 8 electronic newsletters, featuring content related to the risks associated with HPV and the benefits of the vaccination series, as well as identifying barriers to vaccination and strategies to overcome these barriers, were delivered. A separate, private Facebook group with five or more posts each week, that was facilitated by the research staff, was also offered to provide relevant information, to build social support for completing the full vaccination series, and to create favorable social norms around HPV prevention through vaccination. The control intervention was matched in duration and contact schedule to the HW intervention, but did not include weight management content or any information on physical activity or healthy diet, and these participants were not provided with any monitoring devices.

### Measures

All measures were obtained at baseline and immediately after intervention (9 weeks after baseline) unless otherwise noted. Self-report measures were administered in an online questionnaire format with direct data entry by the participants.

#### Anthropometric Data

Body weight was measured to the nearest 0.1 kg in light clothing without shoes using a digital scale (Tanita BWB 800, Arlington Heights, IL). Height was measured to the nearest 0.1 cm at baseline only using a stadiometer. Body mass index (BMI) was calculated as weight (kg)/height (m^2^).

#### Behavioral Weight Control Practices

Behavioral weight control practices were evaluated with a 28-item checklist that assessed both appropriate behavioral weight management strategies (eg, weigh yourself, record food intake, increase exercise levels; *n* =23) and inappropriate weight management strategies (eg, smoke cigarettes, take diet pills, avoid food for 24 hours; *n* =5). The inventory asks individuals to indicate whether they have engaged in the specified behaviors over the previous month and can be examined as either the total number of healthy (or unhealthy) practices endorsed or by examining specific behaviors. The appropriate or healthy weight management items were developed for use in the Look AHEAD trial, and practices identified in the measure have been associated cross-sectionally with lower adiposity [48] and were predictive of long-term weight maintenance [49]. The current study adapted the measure to include items assessing unhealthy or inappropriate weight control strategies (see Multimedia Appendix 1). Change in reported use of weight control practices from baseline to follow-up was the primary focus of the current study, with specific interest in increases in appropriate weight management practices (considered a positive change) and in inappropriate weight management behaviors (considered an iatrogenic, negative change).

#### Sociodemographic Information

Demographic characteristics (ie, age, sex, race, and academic year) were self-reported on the baseline questionnaire.

#### Intervention Engagement

Participant engagement in the HW intervention was assessed by objectively tracking interaction with each platform used and device provided. Engagement data on the newsletters were obtained from MailChimp metrics, which provide information on who opened each newsletter. If a participant opened the newsletter, he or she was considered to have engaged in the newsletter component. If the participant liked or commented on a Facebook post, he or she was considered to have engaged in that platform. In addition, the total number of Facebook likes and comments by each participant were tallied.

Fitbit Zip and Wi-Fi scale usage data were also obtained. The number of students using the devices in a given week was tracked. A participant was considered to have used the Fitbit Zip and Aria scale in a given week if at least one day of data from each respective device was available for the individual in that week. In addition to categorical data on weekly use, the absolute number of days a participant used the Aria scale and Fitbit Zip during the study period and within each week was also tracked.

#### Treatment Satisfaction

After the intervention, participants were asked how useful they found the program and how likely they were to recommend the program to a friend or family member; responses were recorded using a 5-point Likert-type response format. Participants were also asked to rate satisfaction with specific elements of the intervention, such as number of lessons, postings, and the devices.

### Statistical Analyses

Statistical analyses were conducted using SPSS version 22.0 for Windows (IBM Corp., Armonk, NY). Descriptive statistics were calculated for all baseline and engagement measures. Baseline comparisons between conditions were made using the independent *t* test for continuous variables and the chi-square analysis for categorical variables. Two control participants did not complete the postintervention questionnaires (although weight data were collected postintervention for those participants). Therefore, intent-to-treat analyses (with the last value carried forward) of the numbers of appropriate and inappropriate weight control practices reported were compared between conditions. Primary outcomes (body weight and total number of behavioral weight control strategies) were examined with repeated-measures analysis of variance (with time point as the within-participant variable and group as the between-participant variable). Individual univariate comparisons between conditions for individual behavioral weight control strategies reported post-intervention were conducted using the chi-square analysis. Analysis of treatment satisfaction measures aggregated agree, somewhat agree, and strongly agree responses, and combined disagree, somewhat disagree, and strongly disagree, and descriptive data are provided. A *P* value of less than .05 was used to determine statistical significance.

## Results

### Sample Characteristics

All students in the two sections of the targeted health communication class were eligible for and elected to enroll in the study. Students (*n* =58) were upperclassmen who averaged 21.6±2.2 years of age and were predominantly normal weight, although the average baseline BMI was in the upper end of the normal weight range. The majority were white. No significant differences were evident in the baseline characteristics of students in the two conditions. Retention rates at the posttreatment assessment were high, with no significant difference between groups (Table 2).

**Table 2:** Baseline characteristics and retention rates^a^.

Measure		All (*n* =58)	Healthy weight (*n* =29)	Control (*n* =29)	*P*
Age (y)		21.6 ± 2.2	22.1 ± 2.9	21.1 ± 0.8	.08
Female (%)		81	79	83	.74
White (%)		90	83	97	.05
**Academic year (%)**					
	Sophomore	2	3	0	
	Junior	29	14	45	
	Senior	69	83	55	.30
Weight (kg)		67.0 ± 15.8	67.3 ± 11.3	66.6 ± 19.4	.26
BMI^b^(kg/m^2^)		24.0 ± 5.1	24.1 ± 4.3	23.9 ± 5.9	.37
Overweight/obese (BMI ≥ 25 BMI, %)		22	24	21	.75
Retained for follow-up (%)		97	100	93	.15
					

^a^Data are mean ±standard deviation unless indicated by percentage (%).

^b^BMI = body mass index.

### Weight Change

Both groups remained fairly weight stable over the 9-week study period (HW: −0.48±1.9 kg; control: −0.45±1.4 kg), with no significant Group × Time interaction (*P* =.94). Examination of weight changes among individuals who were overweight at the start of the program (22% of sample) revealed no significant differences in weight change between conditions. Overweight students in the HW group (*n* =7) lost 1.8±0.7 kg after 9 weeks compared to 1.4±1.7 kg among overweight students in the control group (*n* =6; *P* =.71).

### Behavioral Weight Control Practices

In contrast to weight, there was a significant Group × Time interaction with respect to the total number of appropriate weight control strategies students reported using in the previous month. An increase in the total number of these strategies was observed at postintervention for those in the HW group (2.1±4.5) versus those in the control group who experienced no significant change (−1.1±3.4; *P*=.003) (Table 3). Specific weight control practices that were higher at posttreatment among those in the HW group compared to those in the control group were self-weighing (*P*=.005), cutting out snacking (*P* =.001), reducing carbohydrate intake (*P* =.02), graphing weight (*P*=.01), reducing calorie intake (*P* =.02), reducing fat intake (*P* =.02), and increasing exercise (*P*=.02). No Group × Time effect for the total number of reported inappropriate weight control strategies was found (*P*=.11), and the absolute number of inappropriate strategies remained low at both time points (Table 3).

**Table 3:** Self-reported use of behavioral weight control strategies^a^.

Measure		All (n=58)	Healthy weight (n=29)	Control (n=29)	*P*
**Appropriate weight control strategies**^b^ **Total number (mean+** **SD**^c^ **)**					
	Pre	8.0 ± 4.1	7.8 ± 4.5	8.3 ± 3.6	
	Post	8.5 ± 3.6	9.9 ± 3.1	7.1 ± 3.6	.003^d^
**Inappropriate weight control strategies**^e^ **, total number (mean±** **SD)**					
	Pre	0.3 ± 0.7	0.3 ±0.7	0.3 ± 0.6	
	Post	0.3 ± 0.6	0.4 ± 0.7	0.1 ± 0.4	.11

^a^Questionnaire available from Multimedia Appendix 1

^b^Total possible number of appropriate weight control strategies (23).

^c^SD: standard deviation.

^d^Statistically significant difference between groups at *P* <.05; Note: *P* values are for Group × Time interactions.

^e^Total possible number of inappropriate weight control practices (5).

### Intervention Engagement and Treatment Satisfaction

**Figure 1:**
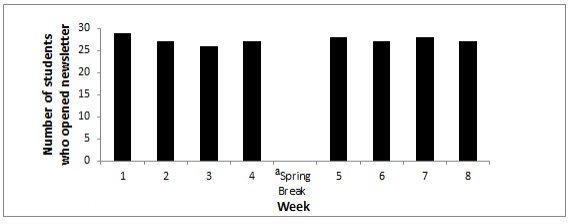
Participant engagement with healthy weight newsletters (N=29).^a^ No electronic newsletters were delivered by the study investigators.

**Figure 2:**
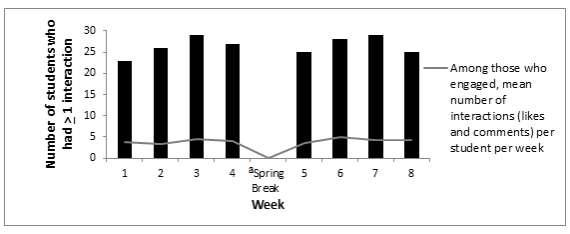
Participant engagement with healthy weight Facebook group (N=29).^a^ No Facebook posts were made by the study investigators.

Electronic newsletters were opened each week by the majority of participants in the HW condition, with all participants (*n* =29) opening the initial weekly newsletter and at least 26 out of 29 participants (90%) opening the newsletters during each subsequent week (Figure 1). The total number of participants who had at least one interaction on the private Facebook page (ie, liked or commented on a post) when study investigators posted ranged from 23 out of 29 (79%) during week 1 to 29 out of 29 (100%) during weeks 3 and 7 (Figure 2). Participants made a total of 862 comments and likes over the intervention period, resulting in an average of 3.3 ±1.4 per person per week, with little variation from week to week except during spring break when there were no posts provided by the intervention to prompt student response (Figure 2). In total, participants averaged 29.5 ±13.0 comments and likes over the course of the intervention, with comments representing the majority of the interactions (26.1±9.7).

**Figure 3:**
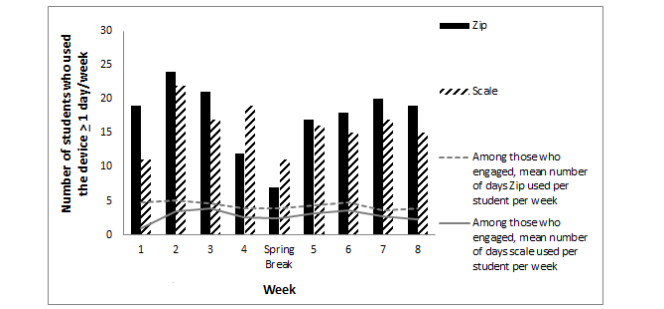
Participant engagement with electronic devices (N=29).

Fitbit Zips were initialized by 26 out of 29 participants (90%) in the HW group, and the number of participants who used the device ranged from 24% (7 out of 29 participants) during spring break to 83% (24 out of 29 participants) during week 2, with students using the Fitbit Zip to record step counts for an average of 23.7±15.2 days across the 9-week observation period (Figure 3). The physical activity tracker was used an average of 2.6 ± 0.9 days per person per week.

Wi-Fi scales were also initialized by 26 out of 29 (90%) HW participants. Two students reported that challenges with the campus Internet were responsible for their failure to initialize. The number of participants who used their Wi-Fi scale ranged from a low of 38% (11 out of 29) during week 1 and spring break to a high of 76% (22 out of 29) during week 2 (Figure 3). On average, students used the Aria scales on 14.1±13.1 days over the intervention period or 1.6±0.7 days a week.

Overall, HW participants rated the intervention positively, with 90% (26 out of 29) indicating they enjoyed it, 86% (25 out of 29) reporting it was helpful, and 83% (24 out of 29) saying that they would recommend the program to a friend. Most participants (26 out of 29; 90%) reported that they were satisfied with the number of lessons, the number of Facebook postings (23 out of 29; 79%), the length of the Facebook postings (24 out of 29; 82%), and the extent of interaction with the study investigators on Facebook (21 out of 29; 72%). Most students also rated the electronic physical activity trackers and Wi-Fi scales positively, with 83% (24 out of 29) rating the trackers and 66% (19 out of 29) rating the Wi-Fi scales as helpful.

## Discussion

### Summary of Principal Results and Comparison to Existing Literature

A technology-mediated, theory-based weight gain prevention intervention targeted at college students that combined the use of Facebook, advanced technological monitoring devices, and electronic newsletters was well accepted by the students. Furthermore, results suggest the approach was efficacious in promoting effective weight management behaviors. The HW intervention led to a significant increase in the number of appropriate weight control behaviors reported by students receiving the intervention relative to the control group. Although no difference in body weight was apparent between conditions over the short intervention period, use of these weight control strategies over time has been shown to predict better weight management among obese individuals [49]. Therefore, continued implementation of these strategies for self-regulating weight may result in differences in weight gain over time between college students who implement more of these weight control behaviors compared with those who do not.

To our knowledge, this study is the first to use both Facebook and wireless monitoring devices to successfully deliver a Web-based weight gain prevention intervention to college students. The generally high level of engagement with the various intervention elements points to the potential this approach has for the target population. In particular, there was very high penetration of the electronic newsletter, with at least 90% of students opening the email each week. Admittedly, opening the newsletter does not indicate that students read the newsletter in detail, nor that they enacted the behavioral strategies for healthy weight management addressed in the newsletter, but it does provide an affirmative indication of treatment receipt. Furthermore, all students interacted with the Facebook group by either posting a comment or “liking” a post or comment. An average of more than three comments and likes were made per person per week, and this high participation level was sustained across the intervention period. This high degree of engagement may reflect how easily accessible these technological tools are to college students and that they are already integrated into their daily lives.

Previous studies have demonstrated that using email [50,51] and Facebook [31,51] to promote healthy lifestyle behaviors and weight loss in college students is feasible; however, the level of engagement with these features was higher in this study than in previous studies [31,50,51]. For example, one study [31] evaluated an 8-week, technology-based weight loss intervention for college students characterized in part by the use of a Facebook group. During this intervention, only 60% of participants posted at least 1 message to their Facebook group and, among those who posted, the average number of posts was approximately 2 messages. In contrast, this study had an average posting frequency of 26 postings over the course of the intervention.

The higher level of email and Facebook engagement observed in this study may be attributable, in part, to the fact that the intervention was integrated into a course for which students received credit. Thus, they may have been more motivated to engage with these technologies than in other studies that did not embed the technology-based intervention within a course [31,50,51]. However, providing course credit or research credit as an incentive for participation in weight gain prevention studies is not uncommon [34,36,52]. Course credit alone does not assure high participation rates; several studies that provided course credit for engaging in the intervention have reported low treatment engagement and/or retention rates [34,52]. Therefore, it is possible that the intervention implemented in this study itself contributed to the high engagement and retention rates. Future weight gain prevention studies in this population should continue to evaluate the effect of providing course credit for participation on both engagement and retention over a longer observation period.

Embedding a weight gain prevention initiative in a college course may also have some particular advantages for a social network–facilitated intervention. Participants in this study may have felt a sense of connectedness as they were enrolled in the same class. In turn, this may have made them more willing to interact via Facebook. Participants in previous Facebook-based studies [31,51,53] were part of ad hoc networks, which may have made some participants reluctant to share their thoughts [54].

This study is the first to provide college students with an electronic physical activity tracker and Wi-Fi scale to promote lifestyle behaviors designed to manage weight, and data on uptake are somewhat promising. Almost all students initialized both devices, and students used the Fitbit Zip an average of 24 days or just under 3.5 weeks across the 9-week observation period. The Wi-Fi scale was used just over 1.5 days a week on average. This was lower uptake than the recommended daily use, which may reflect the fact that students were not selected based on their interest in making lifestyle behavior changes and rather were engaged in the program based on enrollment in a class. Their motivation to monitor their weight and/or their physical activity might have been higher had they volunteered for the program initially based on their interest in weight control, as in the study reported by Napolitano et al [31]. Of interest, the lowest device use was observed in this study over the spring break week. No Facebook interactions from students occurred during this period either. However, no intervention content was delivered by email or Facebook from research staff during this week; hence, there was nothing to prompt students to react. Engagement with social media and device use resumed after the break ended; however, this experience suggests that there is a need to explore ways to facilitate sustained engagement in a technology-mediated weight gain prevention intervention during academic breaks. Data suggest that academic breaks may be a particularly risky time for weight gain for younger children [55], underscoring the importance of addressing this issue. A reasonable initial step would be to continue posting Facebook messages and providing newsletters during breaks, perhaps focusing content on making healthy lifestyle choices while on vacation.

The intervention not only appears to be acceptable but also efficacious in promoting self-regulatory behaviors that have been linked to effective weight control. Noteworthy was the absence of an increase in inappropriate weight control practices following exposure to an intervention that highlighted the need to achieve and maintain a healthy weight, a concern that has been voiced about treatments focusing on weight management among young adults [45]. The development of a pattern of healthy lifestyle behaviors likely to prevent excessive weight gain over time is particularly important in this college-aged population given that these behaviors will likely track into adulthood [1].

As noted, there were no significant differences between conditions in body weight at the end of the short intervention period; both groups were weight stable. However, students were at a healthy weight at baseline, and intervention materials were focused on maintaining a healthy weight; therefore, this is not completely unexpected. Furthermore, the intervention was implemented in the spring semester, a time when people are often making New Year’s resolutions to improve their health behaviors [56]. It is unknown whether weight differences would have emerged if the sample had been followed for a longer period. Most of the limited number of weight gain prevention interventions that have been conducted targeting college students have been short-term interventions (6-15 weeks), and their findings are mixed. For example, Dennis et al [36] observed significant increases in weight among freshmen women engaged in two different SCT-based weight gain prevention interventions, which were delivered through the Internet as part of a course. Providing monetary incentives for remaining weight stable in conjunction with a behavioral intervention resulted in weight changes comparable to those resulting from the behavioral intervention only, without incentives. Average 14-week weight gains were 1.75 and 0.95 kg, respectively. Without a control group in this study, it is unclear whether the interventions attenuated weight gain; evidently, they did not prevent it. In contrast, Levitsky et al [35] included control groups against which to compare two different semester-long interventions characterized by daily weight monitoring with emailed feedback targeted for freshmen women. They found that the control groups gained significantly more weight in each of the two studies (3.1 and 2.0 kg) relative to the experimental groups (0.1 and −0.82 kg), which had negligible gains or modest losses over the 12-week follow-up. Weight gains among those women in the intervention groups were similar to those observed in this study, but the control group participants in the study by Levitsky et al [35] experienced markedly higher weight gain than those in this study. This may reflect the different sample characteristics between the two studies. Levitsky et al [35] enrolled freshmen women exclusively, whereas the sample population in this study was comprised almost entirely of upperclassmen and included both men and women. The rate of weight gain among college students can vary, and freshmen typically gain weight at a higher rate than upperclassmen [7,8,57], which could explain, in part, the difference in weight gains in the control groups observed between the study by Levitsky et al [35] and this study. Indeed, even differences in the timing of the data collection can result in different weight gain trajectories; the first semester of freshman year appears to have the greatest weight gains [9].

This study followed upperclassmen during the spring semester, which perhaps accounts for the minimal weight gain in the control group over the 9-week period. In one of the few longer-duration studies, an intervention focused on maintaining a healthy lifestyle that was delivered to first- and second-year college students in small-group seminars produced small weight losses over 2 years [30], whereas the control group gained weight, resulting in a significant net difference of 1.3 kg between the two groups. This suggests that with extended follow-up periods, the effect of weight gain prevention interventions may be more likely to surface. More definitive studies are required to determine when interventions need to be delivered for college students and how long they should last to achieve the best weight gain prevention outcomes.

### Study Limitations and Strengths

Limitations of this study must be considered when interpreting the results. First, the sample size was small and relatively homogeneous, limiting the generalizability of the findings. The use of a self-report measure of weight control behaviors is another significant study limitation, as is the short intervention exposure and the limited follow-up period. The risk that contamination between conditions occurred must also be considered since students were enrolled at the same university and were aware of the content being covered in both the interventions as a result of reviewing the consent forms. Confounding due to contamination that resulted in an increased focus on weight management among controls could explain the lack of a weight change difference. However, the failure to find an increase in behavioral weight control strategies among controls suggests contamination across conditions was unlikely. Furthermore, the small number of classes randomized is another limitation of this pilot study and points to the need for a larger study of longer duration.

Nevertheless, there are several notable strengths of this pilot study that fuel enthusiasm for the intervention approach implemented. In what is the first report of a behavioral weight gain prevention program for college students that incorporated social media and mHealth monitoring devices, student engagement was robust and treatment satisfaction was high. Moreover, retention was excellent. The use of objective measures of weight and intervention engagement and assessment of both appropriate and inappropriate weight control behaviors represent additional study strengths. The automated delivery of the electronic newsletters and Facebook posts is an additional advantage that points to the potential to readily bring the intervention to scale should it prove efficacious in larger trials over a more extended period.

### Conclusions

An online social media-based weight gain prevention intervention accompanied by mHealth self-monitoring tools increased appropriate weight control efforts by college students relative to controls and demonstrated no short-term iatrogenic effects, although weight change differences between groups were not apparent over the 9-week observation period. Experiences with the intervention indicate students readily engaged with all the technological platforms implemented and found the intervention acceptable. These preliminary findings support the need for the evaluation of this type of intervention over a longer duration in order to determine whether engagement and adherence, as well as the observed behavioral improvements, can be sustained, and in turn, positively affect weight outcomes.

## Appendix

Multimedia Appendix 1 Weight Control Practices Questionnaire.

## Abbreviations

BMI: body mass index
HW: healthy weight
HPV: human papillomavirus
SCT: social cognitive theory
