# Supplementary material for: A Technology-Mediated Behavioral Weight Gain Prevention Intervention for College Students: Controlled, Quasi-Experimental Study
Source: J Med Internet Res. 2016 Jun 13;18(6):e133. doi: 10.2196/jmir.5474 (PMC4923588; doi:10.2196/jmir.5474)
Supplement: Multimedia Appendix 1 [file jmir_v18i6e133_app1.pdf]

<u>Weight Control Practices Questionnaire</u>

For each item on the list:

- **If you did any of these activities <u>during the last month</u> in order to control your weight, check "Yes" and follow the arrow to complete the <u>next column</u> for how many days you did the activity.**
- **If you did not do this, check "no" and go to the <u>next item</u>.**

	Did you do this in the last month?	For how many days did you do this?
1. Weight yourself?	No Yes→	
2. Count fat grams using paper and pencil or an electronic method (such as a smartphone application)?	No Yes→	
3. Cut out between meal snacking?	No Yes→	
4. Eat less high carbohydrate foods like bread or potatoes?	No Yes→	
5. Keep a graph of your weight using paper and pencil or an electronic method (such as a smartphone application)?	No Yes→	
6. Use a very low calorie diet?	No Yes→	
7. Reduce the number of calories you eat?	No Yes→	
8. Smoke cigarettes?	No Yes→	
9. Record what you eat daily using paper and pencil or an electronic method (such as a smartphone application)?	No Yes→	
10. Decrease fat intake?	No Yes→	
11. Go to a weight loss group (such as Weight Watchers or Campus Wellness)?	No Yes→	
12. Eat meal replacements?	No Yes→	
13. Keep a graph of your exercise using paper and pencil or an electronic method (such as a smartphone application)?	No Yes→	
14. Cut out sweets and junk food from your diet?	No Yes→	
15. Increase fruits and vegetables?	No Yes→	
16. Fast or go without food entirely (at least 24 hrs.)?	No Yes→	
17. Count calories using paper and pencil or an electronic method (such as a smartphone application)?	No Yes→	
18. Take diet pills?	No Yes→	
19. Increase your exercise levels?	No Yes→	
20. Eat special low calorie diet foods?	No Yes→	
21. Use home exercise equipment or go to a gym/recreational facility to exercise?	No Yes→	
22. Drink fewer alcoholic beverages?	No Yes→	
23. Monitor your exercise daily using paper and pencil or an electronic method (such as an activity device and/or smartphone application)?	No Yes→	
24. Eat less meat?	No Yes→	
25. Drink regular soda (**not** including diet soda) or **sweetened** fruit drinks?	No Yes→	

26. Drink sports drinks or energy drinks (such as Gatorade or Red Bull)?	No Yes→	
27. Drink **sweetened** tea or specialty coffee drinks (such as cappuccino, latte, or Frappuccino)	No Yes→	
28. **Make** yourself vomit or throw up?	No Yes→	
29. Other (please specify)	No Yes→	
